# Fraction Reduction in Membrane Systems

**DOI:** 10.1155/2014/858527

**Published:** 2014-03-16

**Authors:** Ping Guo, Hong Zhang, Haizhu Chen, Ran Liu

**Affiliations:** ^1^College of Computer Science, Chongqing University, Chongqing 400030, China; ^2^Chongqing Key Laboratory of Software Theory & Technology, Chongqing 400044, China; ^3^Department of Software Engineering, Chongqing College of Electronic Engineering, Chongqing 401331, China

## Abstract

Fraction reduction is a basic computation for rational numbers. P system is a new computing model, while the current methods for fraction reductions are not available in these systems. In this paper, we propose a method of fraction reduction and discuss how to carry it out in cell-like P systems with the membrane structure and the rules with priority designed. During the application of fraction reduction rules, synchronization is guaranteed by arranging some special objects in these rules. Our work contributes to performing the rational computation in P systems since the rational operands can be given in the form of fraction.

## 1. Introduction

Membrane computing (also called P systems) is a branch of natural computing introduced by Pǎun in 1998 which abstracts computing models from the architecture and the functioning of living cells [1]. Membrane computing model takes the living cell as multihierarchical structural regions which are referred to as the membranes [2]. In the compartments defined by membranes there are objects that can evolve to other objects and pass through the membranes. After Pǎun proposed and proved that P systems based on membrane division can solve SAT problems in polynomial time [3], many variants of P systems, including cell-like [4, 5], tissue-like [6], and neural-like ones [7], have been successfully used to design solutions for NP-complete problems. The introductions of the complexity, parallelism, decomposition of membrane, and hierarchical structure can be found in [8, 9].

Based on cell-like P systems which are one kind of common systems in membrane computing, Atanasiu firstly constructs arithmetic P systems to implement arithmetic operations [10]. Reference [11] designs multilayer P systems without priority rules to lower the complexity of the operations. And, in [12], the membrane structure is simplified greatly and efficiency of the computations is also improved owing to arithmetic operations being performed in a single membrane without priority rules. Furthermore, multimembrane P systems are constructed for signed arithmetic operations [13] and the operational range of P system can be extended to the whole integer field. In [14] arithmetic expression is evaluated with primary arithmetical operations implemented in single membranes. Reference [15] proposes an algorithm and builds expression P systems without priority rules for evaluating arithmetic expression. And [16] implements primary arithmetic operations of fractions in P systems and builds a bridge between rational numbers and membrane computing. In cell-like P systems, the operands of the arithmetic operations are represented by multiset, which is composed by the objects and their cardinalities. The rational number can be given by the form of fraction, whose numerator and denominator can be represented by multisets, respectively, so the fraction can be a bridge for implementing the calculations of the rational numbers in P systems.

However, [16] has not further processed the computation results which need to be reduced to lighten the load of the subsequent fraction computations. This paper proposes a suitable method of fraction reduction and implements it in P systems. The rest of this paper is organized as follows: Section 2 introduces cell-like P systems, and Section 3 proposes and proves the fraction reduction method. In Section 4, based on cell-like P system, the rules for implementing fraction reduction are described in detail with the membrane structure designed. The conclusions are drawn in the final section.

## 2. Foundations

### 2.1. Cell-Like P Systems

Our work in this paper is based on cell-like P systems, and such system (of degree *m* ≥ 1) can be defined formally as [1, 2]

(1) $$\begin{array}{c} \Pi =\left(O,\mu ,\omega {}_{1},\ldots ,\omega_{m},R_{1},\ldots ,R_{m},\rho_{1},\ldots ,\rho_{m},i_{o}\right), \end{array}$$

where

- *O*  is the alphabet of the system. Each symbol represents one kind of object. O* is the finite and nonempty multiset over *O* where *λ* is empty string; *O* ^+^ = O* − {*λ*};
- *μ* is a membrane structure with *m* membrane, labeled by 1, 2, …, *m*;
- *ω*_*i*_  (1 ≤ *i* ≤ *m*) is string over *O* representing the multiset of objects placed in membrane *i*. For example, there are 5 copies of object *a* and 3 copies of object *b* in membrane *i*; then we have *ω*_*i*_ = *a* ^5^ *b* ^3^; *ω*_*i*_ = *λ* means that there is no object in membrane *i*;
- *i*_*o*_ is output region of the system and it saves the final results;
- *R*_1_, *R*_2_, …, *R*_*m*_ are finite sets of possible evolution rules over *O* associated with the regions 1, 2, …, *m* of *μ*. The rules in *R*_*i*_ (1 ≤ *i* ≤ *m*) are of the form *U* → *V*|_*a*_, with *a* ∈ *O*, *U* ∈ *O* ^+^, *V* = (*V*′, *ξ*), or *V* = (*V*′, *ξ*)  *δ*, *V*′ ∈ O* and *ξ* = {here, out, in_*j*_ | 1 ≤ *j* ≤ *m*}: here means the product *V*′ remains in the same region; out means *V*′ goes out of the region and enters into another membrane which includes membrane *i* as its submembrane; and in_*j*_ means *V*′ goes into membrane *j* which is a submembrane of membrane *i*. Specifically, when *ξ* = here, (*V*′, *ξ*) can be abbreviated as *V*′. *δ* is a special symbol not in *O*, and it means that the membrane which includes it will be dissolved and the contents of this membrane will be left in the outer membrane. Object *a* is a promoter in the rule *U* → *V*|_*a*_; this rule can only be applied in the presence of object *a*;
- *ρ*_*i*_ (1 ≤ *i* ≤ *m*) defines a partial order relation among the rules in *R*_*i*_. If *ρ*_*i*_ = {*a* → *b* > *c* → *d*} and both objects *a* and *c* are available, then only *a* → *b* can be applied although the two rules do not compete for any objects.

Beside the above rules, we also consider rules for membrane creation, which is of the form *e* → [_*i*_ *V*]_*i*_, with *e* ∈ *O*, *V* ∈ O*, and *i* is a number from a given list of the labels; the idea is that the object *e* creates a new membrane labeled by *i*, including multiset *V* and associated with evolution rules [17].

In each membrane, rules are applied according to the following principles.

- Nondeterminism. Suppose *n* rules compete for the reactants which can only support *m*  (*m* < *n*) rules to be applied; then the *m* rules are chosen nondeterministically.
- Maximal parallelism. All of the rules that can be applied must be applied simultaneously.

From now on we only deal with cell-like P systems with membrane creation and call them P systems for brevity.

### 2.2. Fraction Arithmetic Operations

Reference [16] discusses how to perform fraction arithmetic operations by P systems based on multiple membranes. In [16], the fraction arithmetical operations are written in the form as

(2) $$\begin{array}{c} \frac{\left(+/-\right)m_{1}}{m_{2}}op\frac{\left(+/-\right)n_{1}}{n_{2}},\;op\in \left\{+,-,\times ,÷\right\}, \end{array}$$

where *m*_1_, *m*_2_, *n*_1_, and *n*_2_ are all integers; *m*_2_ > 0, *n*_2_ > 0, *m*_1_ ≥ 0, and *n*_1_ ≥ 0.

Fraction operands are converted into the format which the integer arithmetic requires when the operation is processed. The process of initialization makes the fraction operand be represented in a unified form and it simplifies the operation rules since different operands can be represented by the same objects in the P systems. After initialization, [16] designs four kinds of fraction arithmetic P systems to implement primary arithmetic operations of fractions (namely, addition, subtraction, multiplication, and division).

The computation results obtained by the systems in [16] are not in reduced form. So they are required to be processed further for lightening the load of the subsequent fraction computations. However, the current methods for fraction reductions are not available in P systems. In this paper, we propose a method of fraction reduction and discuss how to carry it out in cell-like P systems.

## 3. A Method for Fraction Reduction

The goal of fraction reduction is to obtain the simplest fraction, and it means the numerator and denominator are coprimes. Generally, fractions can be reduced by the following methods.

- Numerator and denominator are divided by the prime factors that they share until their common factor is 1.
- Numerator and denominator are divided directly by their greatest common factor.

These two methods are simple, but both of them are not suitable for being implemented in P systems owing to the following.

- For the first one, we need to enumerate the primes, such as 2, 3, 5,7,…, to find out the common prime factors of the numerator and denominator. This method involves large calculation and cannot be processed in parallel. If it is implemented in P system, a rule or several rules should be designed for testing whether a prime is their common factor. It means that the more prime factors they share, the more rules are designed and the more complex membrane structure is.
- For the second one, the greatest common factor of the numerator and denominator should be calculated by Euclidean algorithm, but it cannot be performed efficiently in P systems.

For designing a set of generally universal rules to implement fraction reduction in P systems, we present a new fraction reduction method, based on which the designed system works independently on the size of the input. In this section, some theories on the new method are given and the corresponding algorithm is proposed subsequently with its correctness ensured by the present theories.

### 3.1. The Principles for Fraction Reduction

Assume that we have integers *m*, *n* (0 < *n* < *m*) and let *n*_0_ = *m*, *n*_1_ = *n*, the sequences {*a*_*i*_}, {*n*_*i*_}, and {*k*_*i*_} can be constructed as

(3) $$\begin{array}{c} a_{i}=\frac{n_{i}}{n_{i-1}}=\frac{1}{k_{i}+\left(n_{i+1}/n_{i}\right)},\;0\leq n_{i+1}<n_{i},\;i\geq 1, \end{array}$$

where, for *i* ≥ 1,

(4) ki=ni−1div⁡ni,ni+1=ni−1mod⁡ni.

For {*n*_0_, *n*_1_, …, *n*_*ρ*_}, we have the following.

**Theorem 1**

Integer sequence {*n*_0_, *n*_1_, …, *n*_*ρ*_} is monotone decreasing, and there is an integer *v* > 0, such that *n*_*v*_ = 0.

**Proof**

(i) Obviously, {*n*_0_, *n*_1_, …, *n*_*ρ*_} is monotone decreasing according to the procedure of the construction.

(ii) Assume that *n*_*t*_ is the minimum in {*n*_0_, *n*_1_, …, *n*_*ρ*_} and *n*_*t*_ > 0. From the construction, we have

(5) $$\begin{array}{c} n_{t+1}=n_{t-1}\mathrm{mod}n_{t}. \end{array}$$

It is easy to see that 0 ≤ *n*_*t*+1_ < *n*_*t*_. According to the assumption, we obtain *n*_*t*+1_ = 0 and let *v* = *t* + 1, namely, *n*_*v*_ = 0.

From (3), we have

(6) at=ntnt−1=1kt+(nt+1/nt)=1kt,ai=1ki+ai+1, 0≤i<t−1.

The sequence {*f*_*i*_} can be constructed as follows:

(7) ft+1=1,ft=kt,ft−1=kt−1×ft+1.

Generally,

(8) $$\begin{array}{c} f_{i}=k_{i}\times f_{i+1}+f_{i+2},\;1\leq i<t-2. \end{array}$$

So,

(9) at=1ft,at−11kt−1+at=1kt−1+(1/ft)=ftkt−1×ft+1=ftft−1,at−21kt−2+at−1=1kt−2+(ft/ft−1)=ft−1kt−2×ft−1+ft=ft−1ft−2.

Generally, for 0 < *i* < *t*,

(10) ai=1ki+ai+1=1ki+(fi+2/fi+1)=fi+1ki×fi+1+fi+2=fi+1fi.

Specifically,

(11) $$\begin{array}{c} a_{1}=\frac{1}{k_{1}+a_{2}}=\frac{f_{2}}{k_{1}\times f_{2}+f_{3}}=\frac{f_{2}}{f_{1}}. \end{array}$$

**Theorem 2**

*f*_1_ and *f*_2_ are coprimes, and *f*_2_/*f*_1_ is the simplest proper fraction of *n*/*m*.

**Proof**

let *ξ* be the common factor of *f*_1_ and *f*_2_. According to the construction of {*f*_*i*_}, we can obtain *f*_1_ = *k*_1_ × *f*_2_ + *f*_3_, so *ξ* is also the factor of *f*_3_. Similarly, *ξ* is the common factor of *f*_*t*−1_ and *f*_*t*_. It means that there are *ξ*_1_ and *ξ*_2_, such that *f*_*t*−1_ = *ξ* × *ξ*_1_ and *f*_*t*_ = *ξ* × *ξ*_2_. According to the definition of *f*_*t*−1_, we have

(12) $$\begin{array}{c} {f_{t-}}_{1}=\xi \times \xi_{1}=k_{t-1}\times \left(\xi \times \xi_{2}\right)+1. \end{array}$$

That is,

(13) $$\begin{array}{c} \xi \times \left(\xi_{1}-k_{t-1}\times \xi_{2}\right)=1. \end{array}$$

Namely, *ξ* is the factor of 1. Hence, *ξ* = 1. So *f*_1_ and *f*_2_ are coprimes.

According to the construction of {*a*_*i*_} and (11), we have *f*_2_/*f*_1_ = *a*_1_ = *n*/*m*, so *f*_2_/*f*_1_ is the simplest proper fraction of *n*/*m*.

The proofs of the above theories show that the proposed method is feasible for fraction reduction; namely, the simplest proper fraction can be obtained by this method for any fraction.

### 3.2. The Algorithm for Fraction Reduction

Assume that we want to reduce the fraction *n*/*m* (0 < *n* < *m*); from the discussion in Section 3.1, the procedure for fraction reduction can be described as follows:

- input *n*, *m*  (0 < *n* ≤ *m*);
- compute {*n*_*i*_}, {*k*_*i*_}, *i* = 2, 3,…*u*, and *n*_1_ = *n*, *n*_0_ = *m*;
- compute {*f*_*i*_}, *i* = *t*, *t* − 1,…, 1;
- output *f*_1_,*f*_2_.

We can present an algorithm for fraction reduction in Algorithm 1.

In this algorithm, the complexity of the algorithm is *O*(1) when *m* is a multiple of the *n*. Generally, for sequence {*n*_*i*_}, we know *n*_*t*+1_ = 0 if the algorithm performs mod operation for *t* times. Comparing sequence {*n*_*i*_} with Fibonacci sequence {*F*_*i*_}, we have *F*_0_ = 1 ≤ *n*_*t*_ and *F*_1_ = 1 ≤ *n*_*t*−1_. And *n*_*k*_ ≥ *n*_*k*+1_ + *n*_*k*+2_ can be obtained due to *n*_*k*_ mod *n*_*k*_ + 1 = *n*_*k*_ + 2  (0 ≤ *k* ≤ *t* − 1). So *n*_*k*_ ≥ *F*_*t*−*k*_ can be concluded by mathematical induction. Furthermore, we can obtain *m* = *n*_0_ ≥ *F*_*t*_ and *n* = *n*_1_ ≥ *F*_*t*−1_. That is to say, *n* must be not less than *F*_*t*−1_ if our algorithm performs mod operation for *t* times and vice versa. We have $F_{t-1}\geq {\left(1.618\right)}^{t}/\sqrt{5}$ according to the feature of Fibonacci sequence; namely, $n\geq {\left(1.618\right)}^{t}/\sqrt{5}$ and $t\leq \text{lo}{\text{g}}_{1.618}(\sqrt{5}n)$, so the complexity of the algorithm is *O*(log⁡⁡*n*) in the worst case.

## 4. Fraction Reduction in P Systems

In this section, a kind of P systems is designed for fraction reduction based on the algorithm proposed in Section 3.2.

### 4.1. The P Systems for Fraction Reduction

The P systems for fraction reduction can be defined as the form of (1) given in Section 2.1, where:

- *O* is the (finite and nonempty) alphabet of objects which occur in the rules in the designed P system;
- *μ* is the structure of the system and it can be decided by the rules presented subsequently;
- *i*_*o*_ = 1, and it means that the final result can be found in membrane 1 when the whole system halts.

**Figure 1:**
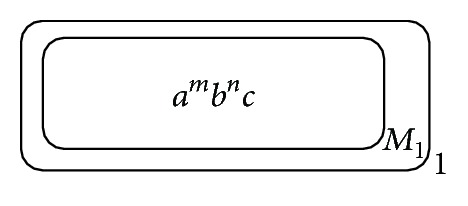
The initial configuration of P system for fraction reduction.

Figure 1 describes the initial configuration of this P system: membrane 1 is responsible for keeping the final results and dissolving the other objects coming from membrane *M*_1_, while fraction reduction is processed in membrane *M*_1_. Except the two membranes, other membranes will be dynamically created during the reduction, and the rules in the new membranes are the same as the ones in membrane *M*_1_. In Figure 1, objects *a* and *b* are used to label the denominator and numerator, respectively; *m* and *n*, which are the cardinalities of *a* and *b*, represent the absolute value of the denominator and numerator, respectively; object *c* is used to trigger the rules in membrane *M*_1_.

**Figure 2:**
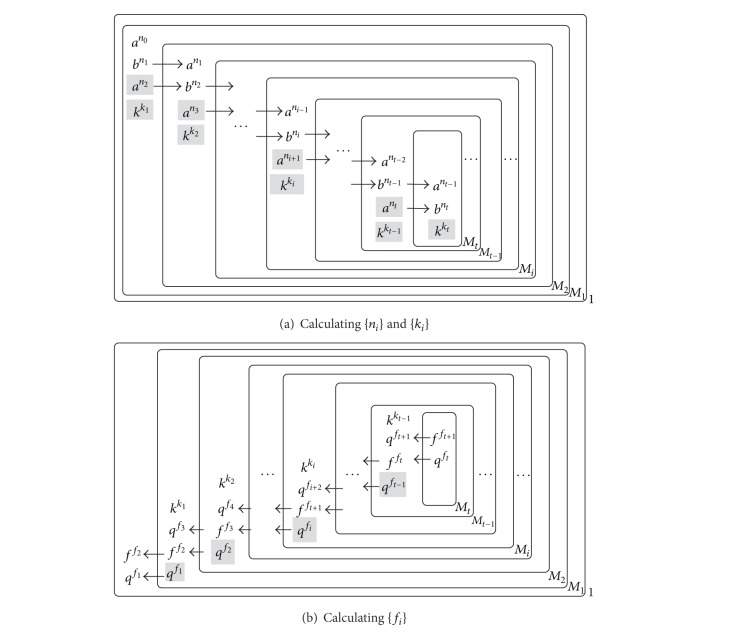
Schematic diagrams for the algorithm in Section 3.2 being implemented by the P systems.

The algorithm proposed in Section 3.2 can be implemented by the P system as shown in Figure 2. In Figure 2, membrane *M*_1_ and the created membranes are responsible for computing {*n*_*i*_}, {*k*_*i*_}, and {*f*_*i*_}. These membranes are nested one by one: the new membrane *M*_2_ is created in membrane *M*_1_, and another new membrane *M*_3_ is created in membrane *M*_2_. Finally, membrane *M*_*t*_ is created in membrane *M*_*t*−1_ (*t* is decided in Section 3.2).

As shown in Figure 2(a), the procedure of calculating {*n*_*i*_} and {*k*_*i*_} is as follows (the cardinalities of the objects are the items in {*n*_*i*_} and {*k*_*i*_}).

- In membrane *M*_1_, multiset *a* ^*n*_0_^ is consumed with new multisets *a* ^*n*_2_^ and *k* ^*k*_1_^ produced by applying several rules. Furthermore, *a* ^*n*_2_^ and *b* ^*n*_1_^ are sent into membrane *M*_2_, and *k* ^*k*_1_^ is kept in *M*_1_.
- When *a* ^*n*_2_^ and *b* ^*n*_1_^ are sent into *M*_2_, they are converted to *b* ^*n*_2_^ and *a* ^*n*_1_^, respectively. In membrane *M*_2_, *a* ^*n*_1_^ is consumed with new multisets *a* ^*n*_3_^ and *k* ^*k*_2_^ produced. Furthermore, *a* ^*n*_3_^ and *b* ^*n*_2_^ are sent into membrane *M*_3_, and *k* ^*k*_2_^ is kept in *M*_2_.
- Generally, in membrane *M*_*i*_  (2 ≤ *i* ≤ *t* − 1), multisets *a* ^*n*_*i*_^ and *b* ^*n*_*i*−1_^ coming from *M*_*i*−1_ are transformed to *b* ^*n*_*i*_^ and *a* ^*n*_*i*−1_^, respectively. And then *a* ^*n*_*i*−1_^ is consumed with the new multisets *a* ^*n*_*i*+1_^ and *k* ^*k*_*i*_^ produced. Furthermore, *a* ^*n*_*i*+1_^ and *b* ^*n*_*i*_^ are sent into membrane *M*_*i*+1_, and *k* ^*k*_*i*_^ is kept in *M*_*i*_.
- Finally, *a* ^*n*_*t*_^ and *b* ^*n*_*t*−1_^ leave membrane *M*_*t*−1_ and they are transferred to *b* ^*n*_*t*_^ and *a* ^*n*_*t*−1_^, respectively, after arriving in membrane *M*_*t*_. In membrane *M*_*t*_, *a* ^*n*_*t*−1_^ is consumed with the *k* ^*k*_*t*_^ produced.

As shown in Figure 2(b), the procedure of calculating {*f*_*i*_} is as follows (the cardinalities of the objects are the items in {*f*_*i*_}).

- In membrane *M*_*t*_, multiset *f* ^*f*_*t*+1_^  (*f*_*t*+1_ = 1) is produced and *k* ^*k*_*t*_^ (*k* ^*k*_*t*_^ is kept in membrane *M*_*t*_ previously) is transformed to *q* ^*f*_*t*_^ (here, *f*_*t*_ = *k*_*t*_). Then, *f* ^*f*_*t*+1_^ and *q* ^*f*_*t*_^ are sent into membrane *M*_*t*−1_.
- When *f* ^*f*_*t*+1_^ and *q* ^*f*_*t*_^ are sent into *M*_*t*−1_, they are converted to *q* ^*f*_*t*+1_^ and *f* ^*f*_*t*_^, respectively. In membrane *M*_*t*−1_, *q* ^*f*_*t*+1_^ and *k* ^*k*_*t*−1_^ (*k* ^*k*_*t*−1_^ is kept in membrane *M*_*t*−1_ previously) are consumed with multisets *q* ^*f*_*t*−1_^ produced. Then, *q* ^*f*_*t*−1_^ and *f* ^*f*_*t*_^ are sent into membrane *M*_*t*−2_.
- Generally, membrane *M*_*i*+1_ sends *q* ^*f*_*i*+1_^ and *f* ^*f*_*i*+2_^ into membrane *M*_*i*_. *q* ^*f*_*i*+1_^ and *f* ^*f*_*i*+2_^ are transformed to *f* ^*f*_*i*+1_^ and *q* ^*f*_*i*+2_^ after arriving in membrane *M*_*i*_. Then *q* ^*f*_*i*+2_^ and *k* ^*k*_*i*_^ (*k* ^*k*_*i*_^ is kept in membrane *M*_*i*_ previously) are consumed with new multisets *q* ^*f*_*i*_^ produced.
- When membrane *M*_1_ sends *q* ^*f*_1_^ and *f* ^*f*_2_^ into membrane 1, the cardinalities of objects *f* and *q* compose the result of the reduction, namely, *f*_2_/*f*_1_.

For convenience, we have some conventions in the rest of the paper as follows.

- The rules should have priority, and they are described as the form (*U* → *V*, *φ*), where *U* → *V* is rewritten rule, and *φ* indicates the priority. The smaller value *φ* is set, higher priority the corresponding rule will have. When *φ* = 1, the corresponding rule will have the highest priority. For example, there are two rules in membrane *M*_1_ : *r*_1_: (*ab* → *x*, 1) and *r*_2_: (*ay* → *ad*, 2), and the priority of *r*_1_ is higher than *r*_2_, so the *r*_1_ will be applied firstly when both of them can be applied.
- The created membranes named *M*_2_, *M*_3_, …, and *M*_*t*_ and the rules in all of them are the same as the ones in membrane *M*_1_.
- The objects appearing in the rest of the paper have the same meaning, so they will not be explained any more once they are introduced previously.

### 4.2. The Rules for Fraction Reduction

In this subsection the rules in the P systems for fraction reduction will be discussed in detail. There are two kinds of rules: one is in membrane 1 and the other is in membranes *M*_1_, *M*_2_, *M*_3_,…, and *M*_*t*_.

According to Section 3, we know that the fraction reduction mainly includes calculating {*n*_*i*_}, {*k*_*i*_}, and {*f*_*i*_}. So membrane *M*_1_ and the created membranes *M*_2_, *M*_3_,…, and *M*_*t*_ should carry out the computations including division (for calculating {*n*_*i*_} and {*k*_*i*_}), multiplication and addition (for calculating {*f*_*i*_}).

#### 4.2.1. The Rules in Membrane *M*_1_

*(i) Calculating *{*n*_*i*_}* and *{*k*_*i*_}. Firstly multiset *a* ^*m*^ *b* ^*n*^ *c* is put in membrane *M*_1_. In this membrane and the created membranes, object *c* evolves to *y* for controlling the division which is used to calculate {*n*_*i*_}, {*k*_*i*_}, and objects *a* and *b* label the denominator and numerator: in membrane *M*_1_, objects *a* and *b* label *n*_0_ and *n*_1_, respectively; in membrane *M*_2_, objects *a* and *b* label *n*_1_ and *n*_2_, respectively; in membrane *M*_3_, objects *a* and *b* label *n*_2_ and *n*_3_, respectively; …; in membrane *M*_*t*_, objects *a* and *b* label *n*_*t*−1_ and *n*_*t*_, respectively.

The rules for calculating {*n*_*i*_} and {*k*_*i*_} should include

(14) r1:(ab⟶x,1),  r2:(c⟶y,1),r3:(ay⟶ad,2),  r4:(x⟶b|d,3),r5:(d⟶kc,4),  r6:(by⟶beg,2),r7:(e⟶[i]i|b,3),  r8:(g⟶h,3),r9:(x⟶(ab,in)|h,4),  r10:(b⟶(a,in)|h,4),r11:(h⟶(c,in),5),  r12:(y⟶kze,3),r13:(x⟶λ|z,3),  r14:(z⟶λ,4).

If *m* > *n*, *r*_1_, *r*_2_, *r*_3_, *r*_4_, and *r*_5_ should be applied in the order of {*r*_1_, *r*_2_} → *r*_3_ → {*r*_4_, *r*_5_} (for convenience, the rules *r*_*j*1_, *r*_*j*2_,…, and *r*_*jk*_ will be represented as {*r*_*j*1_  , *r*_*j*2_,…, *r*_*jk*_}, if they can be executed simultaneously). *a* and *b* are consumed by *r*_1_ with *x* produced; it means that the values of the numerator and denominator are subtracted by *n* simultaneously and (*m* − *n*) copies of object *a* will remain. Object *y*, which is produced by *r*_2_, evolves to *d* with the help of *a* by applying *r*_3_. Once *d* occurs, *x* evolves to *b* by applying *r*_4_ and it means that the numerator is restored for the next division. *d* evolves to *kc* by applying *r*_5_. This procedure may be repeated for several times. Finally, the cardinality of object *k* represents the quotient of the current division.

If *m* < *n*,  *r*_1_,  *r*_2_,  *r*_6_,  *r*_7_,  *r*_8_,  *r*_9_,  *r*_10_, and *r*_11_ should be applied in the order of {*r*_1_, *r*_2_} → *r*_6_ → {*r*_7_, *r*_8_}→{*r*_9_, *r*_10_, *r*_11_}. *r*_1_ and *r*_2_ are applied as described previously, and (*n* − *m*) copies of object *b* will remain. Object *y*, which is produced by *r*_2_, evolves to *eg* with the help of *b* by applying *r*_6_. By applying *r*_7_, *e* triggers to a new membrane to be created in the current membrane in the presence of object *b* and the new membrane will be used to calculate new items in {*n*_*i*_} and {*k*_*i*_}. In the presence of object *h* which is produced by *r*_8_, *r*_9_, and *r*_10_ will be applied: by *r*_9_, *x* evolves to *ab* (they will be sent into the new membrane), and it means that the numerator and denominator consumed by *r*_1_ will be restored in the created membrane; by *r*_10_, *b* evolves to *a* (*a* will be sent into the new membrane). The applications of *r*_9_ and *r*_10_ mean that the numerator becomes the new denominator and the denominator becomes the new numerator in the new membrane for the next division since the division rules will not be triggered in the case of *m* < *n* (there is multiset *a* ^*n*^ *b* ^*m*^ in the new membrane). Object *c* will be produced and sent into the new membrane by applying *r*_11_.

If *m* = *n*, *r*_1_, *r*_2_, *r*_12_, *r*_13_, and *r*_14_ should be applied in the order of {*r*_1_, *r*_2_} → *r*_12_ → *r*_13_ → *r*_14_. *y* evolves to *kze* by applying *r*_12_. In the presence of *z*, *x* will be dissolved by applying *r*_13_. Then *z* will be dissolved by applying *r*_14_. When *e* occurs in the innermost membrane, it means that the calculations of {*n*_*i*_} and {*k*_*i*_} will be ended.

*(ii) Calculating *{*f*_*i*_}. The calculations of {*n*_*i*_} and {*k*_*i*_} will be terminated when object *s* appears and *b* does not appear in the innermost membrane. At this moment, the operations of multiplication and addition should be triggered to calculate {*f*_*i*_}.

Concerning (10) in Section 3, we know that in membrane *M*_*i*_ in Figure 2(b), the copies of objects *q* and *f* will be produced at several steps. So we can design the rules to realize that multisets *f* ^*f*_*i*−1_^ and *q* ^*f*_*i*_^ can be produced in membrane *M*_*i*−1_ while multisets *f* ^*f*_*i*_^ and *q* ^*f*_*i*+1_^ are produced in membrane *M*_*i*_. Based on this consideration, we design the rules for calculating {*f*_*i*_} and they can be applied in several membranes simultaneously. For example, in membrane *M*_*i*_ in Figure 2(b), several copies of objects *q* and *f* are sent into membrane *M*_*i*−1_ once they are produced and they will trigger the rules in membrane *M*_*i*−1_. It means that the rules in membranes *M*_*i*_ and *M*_*i*−1_ will be applied together at the subsequent steps. Maximal parallelism is implemented in the P systems for fraction reduction.

The rules for the operations of multiplication and addition can be designed as follows:

(15) r15:(e⟶pr,4),  r16:(r⟶(f,out),2),r17:(k⟶(q,out)|p,1),  r18:(p⟶(ojw,out)δ,2),r19:(w⟶(ojw,out),3),  r20:(f⟶(q,out),3),r21:(k⟶k(q,out)|q,3),  r22:(o⟶v|q,3),r23:(qj⟶j(f,out),3),  r24:(v⟶o,4),r25:(k⟶λ|o,4),  r26:(j⟶λ|o,4),r27:  (o⟶δ,5).

Rule *r*_15_ is only applied in the innermost membrane *M*_*t*_ and is responsible for *e* evolving to *pr*. Object *r* evolves to *f* by applying *r*_16_, and it means that 1 is assigned to *f*_*t*+1_ in membrane *M*_*t*_ as shown in Figure 2(b). *f* and *q* represent the numerator and denominator, respectively. Object *k* evolves to *q* and *q* is sent into the outer membrane in the presence of *p* by applying *r*_17_ (it is only applied in membrane *M*_*t*_); it means that *k*_*t*_ is assigned to *f*_*t*_ in membrane *M*_*t*_ as shown in Figure 2(b). Rule *r*_18_ is only applied in membrane *M*_*t*_ and is responsible for sending out multiset *ojw* to the outer membrane *M*_*t*−1_. Simultaneously, membrane *M*_*t*_ is dissolved because of the occurrence of the special symbol *δ* when *r*_18_ is applied.

Except for the rules *r*_15_~*r*_18_, the rest of the rules are available in *M*_*t*−1_, *M*_*t*−2_,…, and *M*_1_. By applying *r*_19_, object *w* evolves to *ojw* and *ojw* is sent into the outer membrane for triggering rules *r*_19_, *r*_22_, and *r*_23_ in the outer membrane. Object *f* coming from the inner membrane evolves to *q* and *q* is sent into the outer membrane by applying *r*_20_. Rules *r*_21_ and *r*_22_ are applied to generate objects *q*, *k*, and *v* both in the presence of object *q* and the new generated object *q* is sent into the outer membrane. Rule *r*_23_ is applied to generate objects *j* and *f* (*f* is sent into the outer membrane). Rules *r*_17_, *r*_20_, *r*_21_, and *r*_23_ are responsible for calculating {*f*_*i*_} as shown in Figure 2(b). Object *v* evolves to *o* by applying *r*_24_ for triggering rules *r*_25_ ~ *r*_27_. Rules *r*_25_ and *r*_26_ are applied to consume objects *k* and *j* completely in the presence of object *o*. Rule *r*_27_ is applied to dissolve the current membranes.

#### 4.2.2. The Rule in Membrane 1

There is only one rule in membrane 1 and it is responsible for keeping the final results and dissolving the objects coming from membrane *M*_1_. The rule can be designed as

(16) $$\begin{array}{c} r_{28}:\left(ojw⟶\lambda ,1\right), \end{array}$$

where objects *o*, *j*, and *w* sent from membrane *M*_1_ are dissolved.

Owing to maximal parallelism, the complexity of the P systems for fraction reduction must be not more than *O*(log⁡⁡*n*).

### 4.3. The Instance for Fraction Reduction

**Figure 3:**
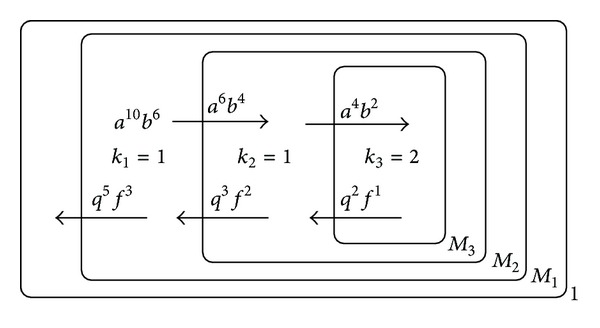
Schematic diagram for reducing 6/10 by the P system.

In this subsection, we will give an instance to show how to implement the fraction reduction in the P system designed previously. For example, 6/10 can be reduced by the P system as shown in Figure 3.

The rules in this P system can be applied as follows.

#### 4.3.1. Initial Configuration

**Figure 4:**
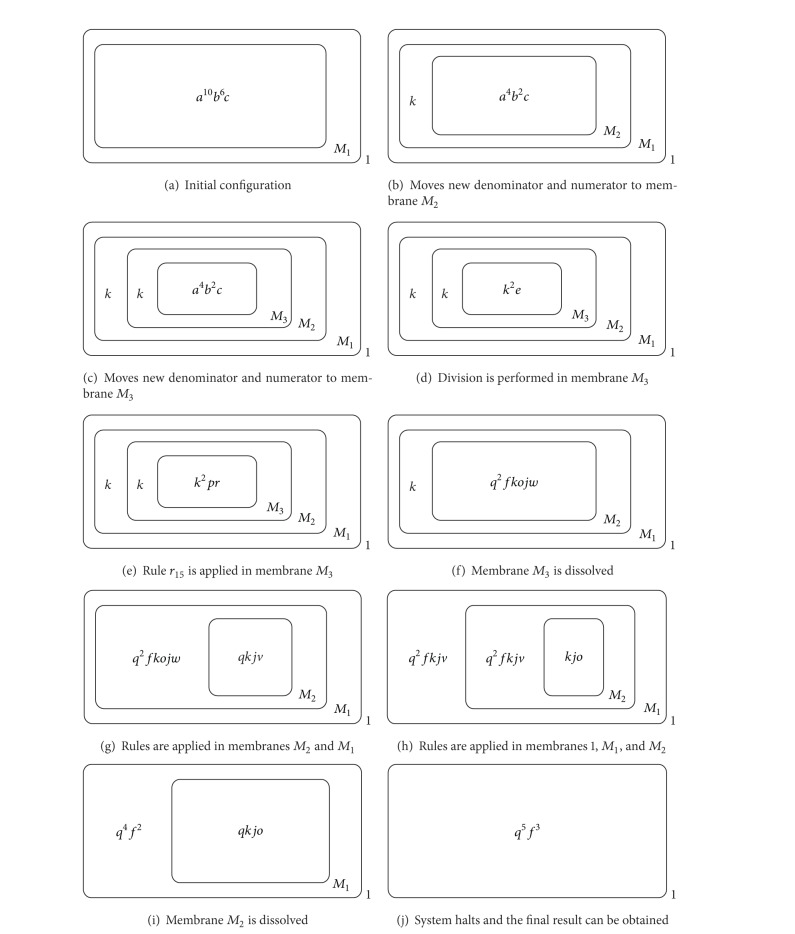
The procedure of reducing 6/10 in the designed P system.

Firstly multiset *a* ^10^ *b* ^6^ *c* is put in membrane *M*_1_, as Figure 4(a) shows: the cardinality of object *a* is 10, and it is the denominator of the fraction; the cardinality of object *b* is 6 and it is the numerator.

#### 4.3.2. Calculating {*n*_*i*_} and {*k*_*i*_}

- In Figure 4(a), rule *r*_1_ is applied 6 times to generate multiset *x* ^6^ until *b* ^6^ is consumed completely, and rule *r*_2_ is applied to generate *y* at the same time. Then only rule *r*_3_ can be applied to generate multiset *ad*. Rule *r*_4_ is applied to restore *x* ^6^ to *b* ^6^ in the presence of object *d*. At the same time, *r*_5_ is applied to generate multiset *ck* and there is multiset *a* ^4^ *b* ^6^ *ck* in membrane *M*_1_. In this case, due to the fact that the cardinality of *a* is less than the one of *b*, the available rules are applied in the order of {*r*_1_, *r*_2_} → *r*_6_ → {*r*_7_, *r*_8_}→{*r*_9_, *r*_10_, *r*_11_} : *r*_1_ is applied 4 times to consume *a* ^4^ completely; *r*_6_ is applied to generate multiset *b* *eg*; by applying *r*_7_, a new membrane is created with the label *M*_2_; by applying *r*_9_, *r*_10_, and *r*_11_, *x* ^4^ and *b* ^2^ are sent into the new created membrane as the new numerator and denominator with object *h* also sent into the new membrane (see Figure 4(b)).
- At this moment, there is multiset *a* ^6^ *b* ^4^ *c* in membrane *M*_2_ (see Figure 4(b)). Hence, the available rules are applied in the order of {*r*_1_, *r*_2_} → *r*_3_ → {*r*_4_, *r*_5_} to generate multiset *a* ^2^ *b* ^4^ *ck*. Owing to the fact that the cardinality of *a* is less than the one of *b*, the available rules are applied in the order of {*r*_1_, *r*_2_} → *r*_6_ → {*r*_7_, *r*_8_} → {*r*_9_, *r*_10_, *r*_11_}: by applying *r*_7_, a new membrane is created with the label *M*_3_; by applying *r*_9_, *r*_10_, and *r*_11_, *x* ^2^ and *b* ^2^ are sent into the new created membrane as the new numerator and denominator with object *h* also sent into the new membrane (see Figure 4(c)).
- There is multiset *a* ^4^ *b* ^2^ *c* in membrane *M*_3_; the available rules are applied in the order of {*r*_1_, *r*_2_} → *r*_3_ → {*r*_4_, *r*_5_} to generate multiset *a* ^2^ *b* ^2^ *ck*. Due to the fact that the cardinality of *a* equals the one of *b*, the available rules are applied in the order of {*r*_1_, *r*_2_} → *r*_12_ → *r*_13_ → *r*_14_ : *r*_12_ is applied to generate multiset *kze*; *r*_13_ is applied 2 times to consume *x* ^2^ completely; then *z* will be dissolved by applying *r*_14_ (see Figure 4(d)).

#### 4.3.3. Calculating {*f*_*i*_}

- There is multiset *k* ^2^ *e* in membrane *M*_3_, and *r*_15_ is applied to generate multiset *pr* (see Figure 4(e)).
- There is multiset *k* ^2^ *pr* in membrane *M*_3_ now. At this moment, *r*_16_ is applied to generate *f* and *f* is sent into the outer membrane. Then *r*_17_ is applied 2 times to generate *q* ^2^ and *q* ^2^ is sent into the outer membrane in the presence of object *p*. Rule *r*_18_ is applied to generate multiset *ojw* *δ* and *ojw* is sent into the outer membrane. Simultaneously, membrane *M*_3_ is dissolved (see Figure 4(f)).
- There is multiset *q* ^2^ *f* *k* *ojw* in membrane *M*_2_. The following rules can be applied in a step: *r*_19_ and *r*_20_ are applied to generate objects *q*, *o*, *j*, and *w* (all of them are sent into the outer membrane); *r*_21_ and *r*_22_ are applied to generate objects *q*, *k*, and *v* both in the presence of object *q* and the new generated object *q* is sent into the outer membrane; *r*_23_ is applied to generate objects *j* and *f* (*f* is sent into the outer membrane) (see Figure 4(g)).
- There are multisets *q* ^2^ *f* *k* *ojw* and *qkjv* in membranes *M*_1_ and *M*_2_, respectively. In membrane *M*_1_, the following rules can be applied in a step: *r*_19_ and *r*_20_ are applied to generate objects *q*, *o*, *j*, and *w* (all of them are sent into the outer membrane); *r*_21_ and *r*_22_ are applied to generate objects *q*, *k*, and *v* both in the presence of object *q* and the new generated object *q* is sent into the outer membrane; *r*_23_ is applied to generate objects *j* and *f* (*f* is sent into the outer membrane). Simultaneously in membrane *M*_2_, the available rules can be applied: *r*_21_ is applied to generate multiset *kq* in the presence of object *q* and the new generated object *q* is sent into the outer membrane; *r*_23_ is applied to generate multiset *fj* and *f* is sent into the outer membrane, and *r*_24_ is applied to generate object *o* at the same time (see Figure 4(h)).
- There are multisets *q* ^2^ *f* *ojw*, *q* ^2^ *fkjv*, and *kjo* in membranes 1, *M*_1_, and *M*_2_, respectively. In membrane 1, rule *r*_28_ is applied to consume multiset *ojw* completely. In membrane *M*_1_, the following rules can be applied in a step: *r*_20_ is applied to generate object *q* (*q* is sent into the outer membrane); *r*_21_ is applied to generate objects *q* and *k* in the presence of object *q* and the new generated object *q* is sent into the outer membrane; *r*_23_ is applied to generate objects *j* and *f* (*f* is sent into the outer membrane); *r*_24_ is applied to generate object *o*. In membrane *M*_2_, rules *r*_25_ and *r*_26_ are applied to consume objects *k* and *j* completely; then membrane *M*_2_ is dissolved after applying *r*_27_ (see Figure 4(i)). The aforementioned rules in membranes 1, *M*_1_, and *M*_2_ are applied simultaneously.
- There are multisets *q* ^4^ *f* ^2^ and *q* *kjo* in membranes 1 and *M*_1_, respectively. In membrane *M*_1_, the following rules can be applied in a step: *r*_20_ is applied to generate object *q* (*q* is sent into the outer membrane); *r*_21_ and *r*_22_ are applied to generate objects *q*, *k*, and *v* both in the presence of object *q* and the new generated object *q* is sent into the outer membrane; *r*_23_ is applied to generate objects *j* and *f* (*f* is sent into the outer membrane). Then there is multiset *kjv* in membrane *M*_1_, so the following rules can be applied: *r*_24_ is applied to generate object *o*; *r*_25_ and *r*_26_ are applied to consume objects *k* and *j* completely; membrane *M*_1_ is dissolved after applying *r*_27_ (see Figure 4(j)).
- There is multiset *q* ^5^ *f* ^3^ in membrane 1 (see Figure 4(j)). At this time, no rules can be applied, so the whole system halts. The cardinalities of *q* and *f* represent the values of the denominator and numerator, respectively, so the final result of reducing 6/10 is 3/5.

## 5. Conclusions

Fraction (rational number) computing is foundational in most of the computing models and systems, and the computation results of the fractions often need to be reduced to lighten the load of the subsequent computations. This paper proposes and proves a new suitable reduction method and implements it in P systems. Furthermore, we give an instance to illustrate how to carry out the fraction reduction effectively in this system. For the fact that the rational number can be given by the form of fraction, whose numerator and denominator can be represented by multisets, respectively, our work will contribute to implementing the computation of the rational numbers in P systems. Further, we will research the signed fraction reduction in P systems and the fraction reduction in the case that the denominator or numerator is 0.

## Figures and Tables

**Algorithm 1:**
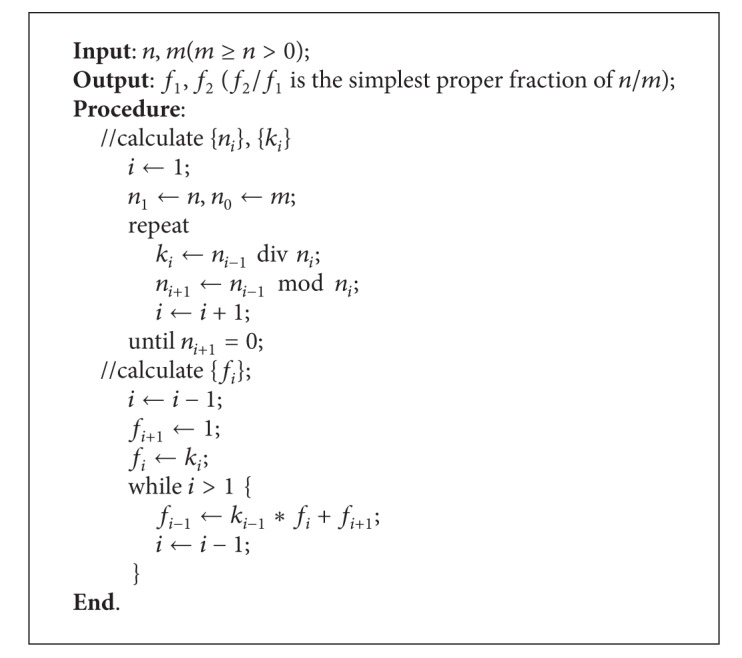
Fraction reduction.
